# Evaluation of MRI characterisation of histopathologically matched lymph nodes and other mesorectal nodal structures in rectal cancer

**DOI:** 10.1007/s00330-025-11361-2

**Published:** 2025-01-21

**Authors:** Miriam Kheira Rutegård, Malin Båtsman, Lennart Blomqvist, Martin Rutegård, Jan Axelsson, Wendy Wu, Ingrid Ljuslinder, Jörgen Rutegård, Richard Palmqvist, Fredrik Brännström, Katrine Riklund

**Affiliations:** 1 Department of Diagnostics and Intervention, Diagnostic Radiology, Umeå University, Umeå, Sweden; 2 Department of Medical Biosciences, Pathology, Umeå University, Umeå, Sweden; 3 Department of Molecular Medicine and Surgery, Karolinska Institutet, Solna, Sweden; 4 Department of Diagnostics and Intervention, Surgery, Umeå University, Umeå, Sweden; 5 Department of Diagnostics and Intervention, Radiation Physics, Umeå University, Umeå, Sweden; 6 Department of Diagnostics and Intervention, Oncology, Umeå University, Umeå, Sweden

**Keywords:** Rectal neoplasms, Magnetic resonance imaging, Neoplasm staging, Lymphatic metastasis, Extranodal extensions

## Abstract

**Purpose:**

To evaluate current MRI-based criteria for malignancy in mesorectal nodal structures in rectal cancer.

**Method:**

Mesorectal nodal structures identified on baseline MRI as lymph nodes were anatomically compared to their corresponding structures histopathologically, reported as lymph nodes, tumour deposits or extramural venous invasion. All anatomically matched nodal structures from patients with primary surgery and all malignant nodal structures from patients with neoadjuvant treatment were included. Mixed-effects logistic regression models were used to evaluate the morphological criteria irregular margin, round shape, heterogeneous signal and nodal size, as well as the combined 2016 European Society of Gastrointestinal and Abdominal Radiology (ESGAR) consensus criteria, with histopathological nodal status as the gold standard.

**Results:**

In total, 458 matched nodal structures were included from 46 patients (mean age, 67.7 years ± 1.5 [SD], 27 men), of which 19 received neoadjuvant treatment. The strongest associations in the univariable model were found for short-axis diameter ≥ 5 mm (OR 21.43; 95% CI: 4.13–111.29, *p* < 0.001) and heterogeneous signal (OR 9.02; 95% CI: 1.33–61.08, *p* = 0.024). Only size remained significant in multivariable analysis (OR 12.32; 95% CI: 2.03–74.57, *p* = 0.006). When applying the ESGAR consensus criteria to create a binary classification of nodal status, the OR of malignant outcome for nodes with positive ESGAR was 8.23 (95% CI: 2.15–31.50, *p* = 0.002), with corresponding sensitivity and specificity of 54% and 85%, respectively.

**Conclusion:**

The results confirm the role of morphological and size criteria in predicting lymph node metastases. However, the current criteria might not be accurate enough for nodal staging.

**Key Points:**

***Question*** *Pretreatment lymph node staging in rectal cancer is challenging, and the ESGAR consensus criteria are not fully validated*.

***Findings*** *Although the ESGAR criteria correlated with malignant outcomes, diagnostic performance in terms of particular sensitivity, but also specificity, was not high*.

***Clinical relevance*** *Accurate nodal staging in rectal cancer is crucial for individual treatment planning. However, this validation of the current ESGAR consensus criteria suggests that these should be used with caution*.

## Introduction

Pelvic magnetic resonance imaging (MRI) is considered the standard of care for rectal cancer staging. Nevertheless, MRI regional metastatic lymph node assessment (N staging) is still associated with limited sensitivity and specificity [1]. Determining the N stage accurately in rectal cancer is important since the presence and extent of metastatic lymph nodes could affect the treatment strategy, including the use of neoadjuvant treatment—particularly in early and intermediate disease [2–4]. Furthermore, mesorectal lymph nodes are difficult to differentiate from tumour deposits (TDs) and even nodular extramural venous invasion (EMVI), which both increase the risk of distant spread and are associated with poorer outcomes compared to malignant lymph nodes alone [5–8]. This is not fully considered in the current TNM staging nor the Swedish or the 2017 European Society for Medical Oncology (ESMO) clinical practice guidelines [3, 4, 8].

In 2021, Zhuang et al conducted a systematic review and meta-analysis to evaluate lymph node staging by MRI using various sizes and morphological standards (including irregular border and heterogeneous signal) [9]. The pooled sensitivity and specificity for N-staging were 73% and 74%, respectively, similar to earlier meta-analyses, and when only considering the five of 36 studies that had an anatomical matching of individual lymph nodes between radiology and the pathology report, the pooled sensitivity and specificity were 55% and 89%, respectively [9–12]. There was no statistically significant difference between size-only criteria and morphological features combined with the different size cut-offs used [9].

The current 2016 MRI criteria from the European Society of Gastrointestinal and Abdominal Radiology (ESGAR) expert consensus panel states that to be considered malignant on MRI, mesorectal nodal structures less than 5 mm in short axis diameter should have three morphological features suspicious for malignancy on T2-weighted images (round shape, irregular border, and heterogeneous signal), nodal structures with a short axis size of 5–8 mm should have two morphological suspicious characteristics and nodal structures with a short axis size of 9 mm or more should be considered malignant regardless of the morphological features present [13]. Although the ESGAR consensus criteria have been used locally in the Netherlands since 2014 and internationally since 2017, they are not fully validated, with round shape being the least studied morphological criterion [9, 13]. In addition, it is unclear how many, if any, of the included studies in the meta-analysis used round shape as a malignant morphological characteristic, and none of them used the size cut-off recommended in the ESGAR criteria [9]. Also, as mentioned earlier, only five of the 36 included studies had an anatomical matching of individual lymph nodes between radiology and the pathology report. In summary, there is a lack of research on the application of the ESGAR criteria in general and, in particular, on a node-by-node basis in rectal cancer MRI staging. There is also a knowledge gap concerning the frequency of TDs and nodular EMVI in rectal cancer, and guidelines on differentiating these entities are missing.

In this study, we aimed to evaluate the performance of the current ESGAR consensus criteria, considering the constituent morphological and size criteria separately, and investigate the optimal combination of criteria for predicting malignant mesorectal nodal structures in rectal cancer patients. We also investigated the association between the comet tail appearance and TDs [14]. This study is based on a prospective cohort study in which individual mesorectal nodal structures were anatomically matched to histopathology.

## Material and method

### Patient population and characteristics

**Fig. 1:**
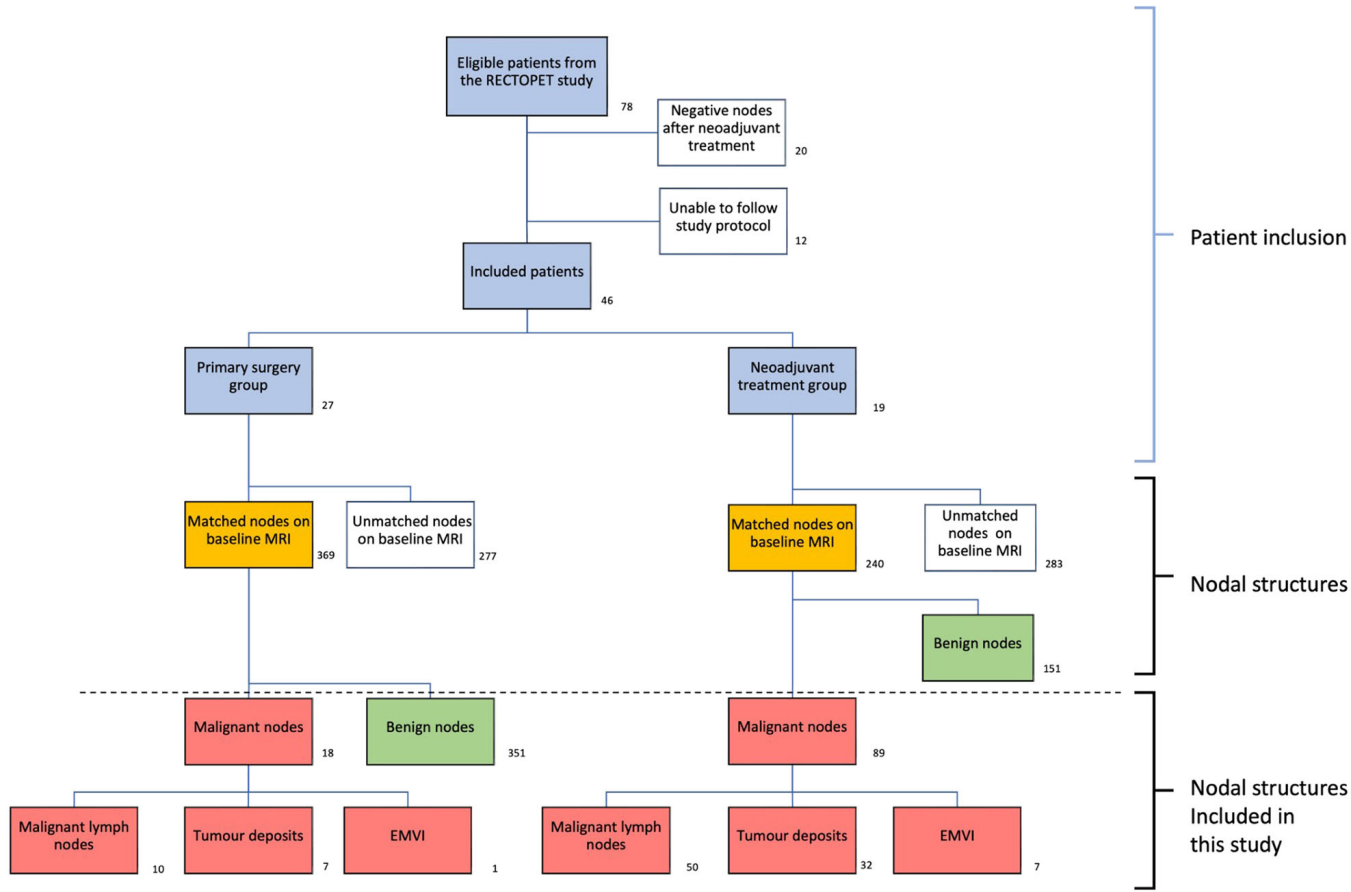
Flowchart of the inclusion process, featuring the final number of included patients, as well as the number and type of anatomically, matched nodal structures according to the histopathological assessment

This study is based on the prospective observational study RECTOPET (rectal cancer trial on PET/CT/MRI—NCT03846882), approved by the local Regional Ethical Review Board and Radiation Protection Committee in 2015 (2015/417-31) with written informed consent as a requirement. All eligible adult patients in Region Västerbotten with biopsy-proven rectal cancer (up to 15 cm from the anal verge) and written informed consent were included consecutively between September 2016 and May 2021, as described elsewhere [15]. The exclusion criteria were contraindications for MRI or contrast-enhanced computerised tomography (CT), other diagnoses than rectal cancer, and emergency surgery. The additional inclusion criterion for this study was the presence of a surgical specimen with an individual anatomical matching of mesorectal nodal structures between radiology and histopathology. This excluded patients who did not undergo curative surgical treatment, patients with incomplete radiology, or a lack of a histopathological assessment according to the study protocol. The radiological evaluations were made on baseline MRI before neoadjuvant treatment. All mesorectal nodal structures were included in patients who had primary surgery, but for patients who received neoadjuvant treatment, only histopathologically proven malignant nodal structures were included (Fig. 1). This was because the malignant nodes’ probability of malignancy at the baseline MRI must be considered all but certain. In contrast, the benign nodal structures could have been malignant before treatment. Mesorectal nodal structures were defined as round or oval structures in the mesorectal fat and along the superior rectal vessels that were assessed as lymph nodes in MRI but histopathologically proved to be lymph nodes, TDs and EMVI in the histopathological assessment.

### ^18^F-fluoro-2-deoxy-d-glucose (FDG)-positron-emission tomography (PET)/CT and FDG-PET/MRI examinations

All included patients in the RECTOPET study had diagnostic FDG-PET/CT and FDG-PET/MRI examinations instead of the conventional diagnostic CT and MRI examinations [15]. In short, the PET/CT was performed by Discovery 690 PET/CT scanner (GE Healthcare), and the PET-MRI by a 3-T SIGNA PET/MRI (GE Healthcare), using standard clinical protocols with the addition of a 3D T1-weighted sequence with 1 mm slice thickness covering the whole pelvic region (Supplemental Table 1). The PET/CT acquisition was initiated 60 min after the injection of 4 MBq/kg [^18^F]FDG, and the PET/MRI scan was initiated 120 min after the same tracer injection. If neoadjuvant treatment was given, a preoperative restaging FDG-PET/CT and FDG-PET/MRI were acquired 6–8 weeks after the end of treatment.

Postoperatively, an MRI of the surgical specimen was performed before the histopathological cutout, and the study pathologist (M.B.) created a histopathological finding-by-finding description for each specimen, as described in detail elsewhere [7]. The individual anatomical matching of nodal structures between MRI and histopathology was made continuously during the inclusion period through consensus meetings between the study radiologist (M.K.R.) and pathologist (M.B.).

### MRI interpretation

All anatomically matched mesorectal nodal structures assessments on baseline MRI were performed approximately one year after the inclusion had ended by the study radiologist (M.K.R. [with six years of experience in pelvic MRI and a specialist in radiology for two years]) blinded to the histopathological findings. The evaluation was made with guidance from a radiologist with thirty years of experience in rectal MRI (L.B.).

Since only the study radiologist assessed all included patients, no inter-reader evaluations could be made. The documented parameters were short axis size, round shape, comet tail appearance, irregular margin, and heterogeneous signal, all assessed in the transaxial plane in a T2-weighted sequence (perpendicular to the primary tumour when applicable). The feature round shape was defined as the ratio between short and long axis sizes equal to one (with a margin of error of 0.05). All anatomically matched nodal structures were assessed as malignant or not according to the 2016 ESGAR consensus criteria [13].

### Statistical analysis

Descriptive statistics was used to evaluate the patient cohort, pathological outcome, and morphological characteristics. Mixed-effects logistic regression models were used to estimate the odds ratios (ORs) and 95% confidence intervals (CIs). The models were fitted with individual-specific random intercepts to account for clustering of nodes within individuals. We included irregular margin, round shape, heterogeneous signal, and short axis size in the multivariable model to investigate the independent associations with the risk of malignancy as a binary outcome. Because of the small sample size, we did not estimate associations with the different types of malignant nodal structures according to the histopathological assessment, i.e. malignant lymph nodes, TDs and nodular EMVI.

We calculated the sensitivity, specificity, and positive and negative likelihood ratios (LRs) for the individual and the combined variables to ascertain their performance in identifying nodal involvement. Non-parametric bootstrapping was used to calculate the CIs for the positive and negative LRs to account for within-patient correlation. In a post hoc subgroup analysis, these analyses were performed to ascertain any performance differences in early/intermediate rectal cancer (defined as clinical stage T3b or less, no involvement of mesorectal fascia, and no presence of EMVI).

The statistical analyses were performed using R statistical computing, version 4.3.0 and the R packages “lme4” and “boot”.

## Results

In our study, 46 patients (27 males and 19 females with a median age of 70 years and an interquartile range of 62–74 years) were included; 27 had primary surgery (the PS group), and 19 received neoadjuvant treatment before surgery (the NT group). Figure 1 depicts the inclusion process for the 78 eligible patients from the RECTOPET study, where twelve patients were excluded due to incomplete imaging or surgical specimens not being processed according to the study protocol, and twenty patients were excluded due to the lack of anatomically matched malignant nodal structures after neoadjuvant treatment. Table 1 demonstrates the clinical data for the included patients.

**Table 1:** Baseline characteristics for the 46 included rectal cancer patients

Clinical data	Total
	*N* = 46
Age (years)	70.0 (62.0–74.0)
Sex	
Male	27 (58.7%)
Female	19 (41.3%)
Tumour height (cm)	7.0 (5.0–11.0)
Clinical tumour stage	
I	20 (43.5%)
II	10 (21.7%)
III	11 (23.9%)
IV	5 (10.9%)
Clinical management	
Upfront surgery	27 (58.7%)
Radiotherapy^a^	12 (26.1%)
Chemoradiotherapy^b^	7 (15.2%)
Operation type	
Anterior resection	23 (50.0%)
Abdominoperineal excision	23 (50.0%)
Pathological (y)pT stage	
T0	1 (2.2%)
T1	2 (4.3%)
T2	12 (26.1%)
T3	30 (65.2%)
T4	1 (2.2%)
Pathological (y)pN stage	
N0	21 (45.7%)
N1	16 (34.8%)
N2	9 (19.6%)

Continuous variables are presented with median and interquartile ranges

^a^ Five Gy for five consecutive days—surgery 8–12 weeks after completion

^b^ Five Gy for five consecutive days followed by 4–6 cycles of chemotherapy (capecitabine and oxaliplatin)—surgery 8–12 weeks after completion

In the 46 included patients, 1169 nodal structures were found at baseline MRI and 1697 at the histopathological cutout; 609 nodal structures were anatomically matched between MRI and histopathology. The unmatched nodal structures were smaller than the matched ones (*p* < 0.001), with a median MRI short axis size and a median histopathological short axis of 2.3 mm (IQR 1.7–3.2 mm) and 1.7 mm (IQR 1.2–2.4 mm), respectively. For the matched nodal structures, the median MRI short axis size and histopathological short axis size were 3.2 mm (IQR 2.4–4.7 mm) and 2.7 mm (IQR 2.0–3.7 mm), respectively (Supplemental Table 2). The proportion of malignant nodal structures was 107 out of 609 (17.6%) among the matched nodal structures and 90 out of 1088 (8.3%) for the unmatched ones.

**Fig. 2:**
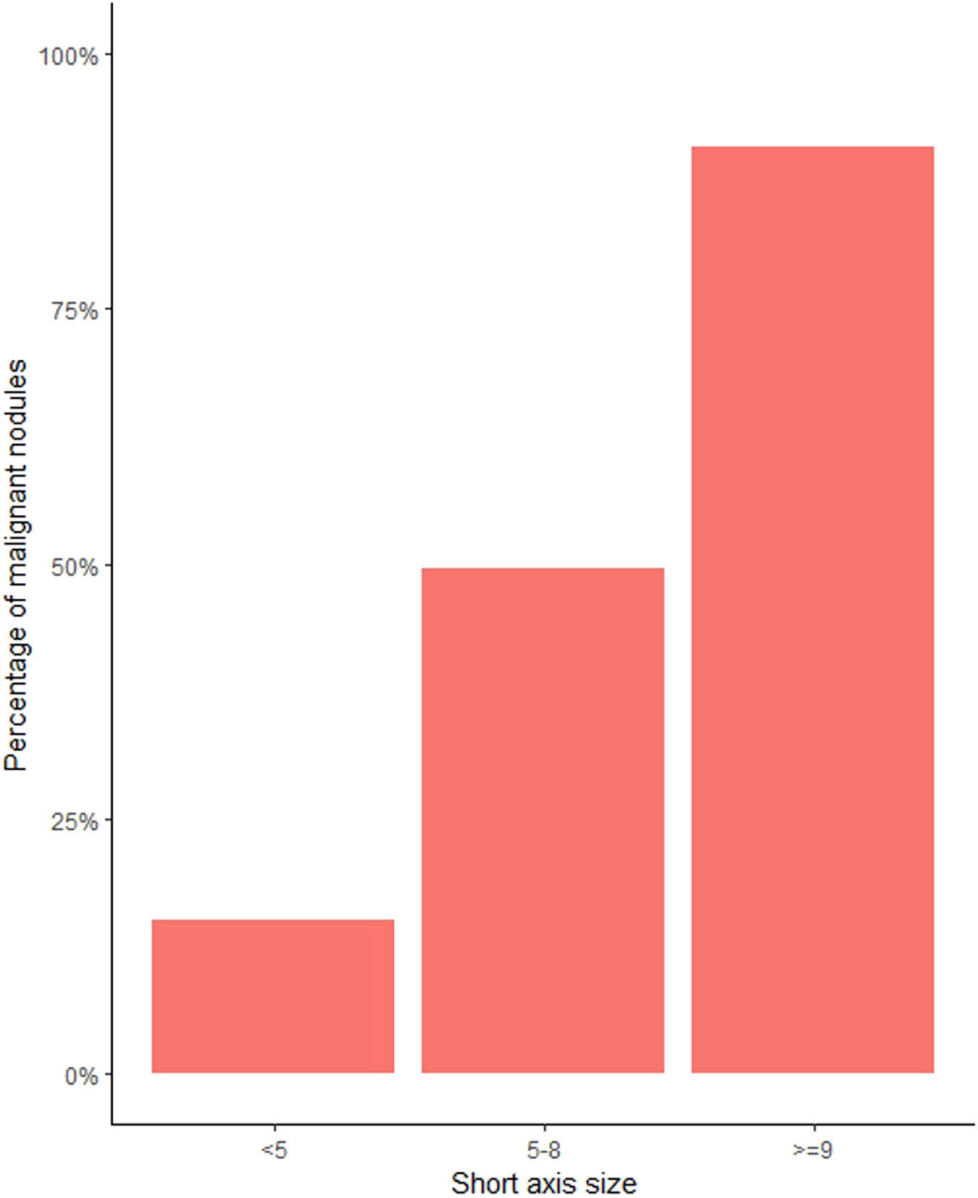
The proportion of malignant nodes for the different short axis size categories used in the 2016 ESGAR consensus criteria

Of the 609 anatomically matched nodal structures, 458 were included in this study since only the malignant nodal structures were included in the group of patients who received neoadjuvant treatment. The proportion of benign and the different types of malignant nodal structures is demonstrated in Fig. 1. In the PS group, 8 of 18 (44%) malignant nodal structures initially assessed as lymph nodes proved to be TDs or nodular EMVI histopathologically. The corresponding number in the NT group was 39 out of 89 (44%). Of the 458 included nodal structures, 357 (78.0%) had an MRI short axis size < 5 mm. The size distribution among the included malignant nodal structures is shown in Fig. 2.

Associations between malignancy and constituent features in the ESGAR consensus criteria were evaluated, and the results are demonstrated in Table 2. The comet tail appearance is not part of the ESGAR consensus criteria but has been proposed as a sign of TDs [14]. This feature was noted in 22 (4.8%) out of the 458 included nodal structures, of which 10 (45.4%) were benign lymph nodes, 8 (36.4%) TDs, 3 (13.6%) malignant lymph nodes and 1 (4.5%) EMVI.

**Table 2:** Univariable and multivariable odds ratios (ORs) with 95% CIs for malignancy for different morphological characteristics in mesorectal nodal structures in rectal cancer

Radiological characteristics	*N*−	*N*+	Univariable model			Multivariable model		
	*n* (%)	*n* (%)	OR	95% CI	*p* value	OR	95% CI	*p* value
Irregular margin								
No	150 (90.9%)	15 (9.1%)	1.00			1.00		
Yes	201 (68.6%)	92 (31.4%)	6.46	1.48–28.15	0.013	1.39	0.23–8.23	0.715
Round shape								
Yes	318 (77.6%)	92 (22.4%)	1.00			1.00		
No	33 (68.7%)	15 (31.3%)	3.59	0.71–18.26	0.123	2.24	0.33–15.10	0.406
Comet tail appearance^a^								
No	341 (78.2%)	95 (21.8%)	1.00					
Yes	10 (45.5%)	12 (54.5%)	5.56	0.75–41.00	0.092			
Heterogeneous signal								
No	142 (91.0%)	14 (9.0%)	1.00			1.00		
Yes	209 (69.2%)	93 (30.8%)	9.02	1.33–61.08	0.024	3.58	0.42–30.58	0.244
Short axis size								
< 5 mm	305 (85.4%)	52 (14.6%)	1.00			1.00		
≥ 5 mm	46 (45.5%)	55 (54.5%)	21.43	4.13–111.29	< 0.001	12.32	2.03–74.57	0.006
Malignant according to ESGAR								
No	297 (85.8%)	49 (14.2%)	1.00					
Yes	54 (48.2%)	58 (51.8%)	8.23	2.15–31.50	0.002			

The individual variables used for the multivariable model are the constituent criteria in the current ESGAR criteria, i.e. irregular margin, round shape, heterogeneous signal, and short axis size

^a^ As a sign of TDs proposed by Lord et al [14]

In the univariable model, statistically significant associations with a histopathologically proven malignancy were found for all the evaluated morphological features except for round shape (OR 3.59 [95% CI: 0.71–18.26], positive LR 1.49, negative LR 0.95, *p* = 0.123) and comet tail appearance (OR 5.56 [95% CI: 0.75–41.0], *p* = 0.092). The strongest associations were seen for short axis size ≥ 5 mm (OR 21.43 [95% CI: 4.13–111.3], positive LR 3.92, negative LR 0.56, *p* < 0.001) followed by heterogeneous signal (OR 9.02 [95% CI: 1.33–61.08], positive LR 1.46, negative LR 0.32, *p* = 0.024). The same pattern was seen when evaluating the PS group only (Supplemental Table 3).

A multivariable model rendered a positive association between the constituent features of the 2016 ESGAR consensus criteria and malignancy, although only short axis size ≥ 5 mm remained statistically significant (multivariable OR 12.32 [95% CI: 2.03–74.57], *p* = 0.006). When applying the 2016 ESGAR consensus criteria to create a binary variable to indicate positive or negative nodal status, the OR of malignancy for lesions with a positive ESGAR status was 8.23 (95% CI: 2.15–31.5], positive LR 3.52, negative LR 0.54, *p* = 0.002), indicating a significantly higher association with malignancy compared to lesions with a negative ESGAR status.

Table 3 shows the sensitivity, specificity, and positive and negative LRs for the different criteria for assessing malignant nodal outcomes. The current ESGAR consensus criteria had a sensitivity, specificity, and positive and negative LR of 54%, 85%, 3.52 and 0.54, respectively. Regarding individual characteristics, heterogenous signal demonstrated the highest sensitivity of 87%, whilst round shape had the highest specificity of 91%.

**Table 3:** The sensitivity, specificity, positive and negative LR with 95% CIs for different criteria to assess malignancy in mesorectal nodal structures in rectal cancer

	Sensitivity	Specificity	Positive LR	Negative LR
Single criterion				
Size ≥ 5 mm	51%	87%	3.92 (2.38–6.96)	0.56 (0.38–0.74)
Irregular margin	86%	43%	1.50 (1.26–1.81)	0.33 (0.18–0.63)
Round shape	14%	91%	1.49 (0.76–2.73)	0.95 (0.85–1.03)
Heterogeneous signal	87%	40%	1.46 (1.25–1.71)	0.32 (0.15–0.58)
Combined criteria				
Size ≥ 5 mm, size < 5 mm with heterogeneous signal	87%	40%	1.46 (1.25–1.71)	0.32 (0.15–0.58)
Size ≥ 5 mm, size < 5 mm with irregular margin	87%	42%	1.51 (1.28–1.83)	0.31 (0.17–0.57)
Size ≥ 5 mm, size < 5 mm with round shape	58%	80%	2.95 (1.98–4.70)	0.52 (0.33–0.73)
Size ≥ 5 mm, size < 5 mm with round shape and irregular margin	54%	83%	3.28 (2.05–5.46)	0.55 (0.36–0.74)
Size ≥ 5 mm, size < 5 mm with either a round shape or irregular margin	91%	39%	1.49 (1.31–1.77)	0.24 (0.11–0.42)
Size ≥ 5 mm, size < 5 mm with round shape and heterogeneous signal	55%	83%	3.28 (2.07–5.24)	0.54 (0.35–0.74)
Size ≥ 5 mm, size < 5 mm with either round shape or heterogeneous signal	90%	38%	1.44 (1.24–1.68)	0.27 (0.12–0.61)
ESGAR consensus criteria	54%	85%	3.52 (2.18–5.85)	0.39 (0.23–0.55)
ESGAR consensus criteria minus round shape^a^	81%	51%	1.66 (1.34–2.11)	0.37 (0.19–0.65)

*ESGAR* European Society of Gastrointestinal and Abdominal Radiology

^a^Defined as a short axis size of < 5 mm with heterogeneous signal and irregular margin, a short axis size of 5–8 mm with either heterogeneous signal or irregular margin, or a short axis size of > 9 mm

For the combined criteria, the combination of short axis size ≥ 5 mm or short axis size < 5 mm and either round shape or irregular margin demonstrated the highest sensitivity of 91%, whereas the ESGAR consensus criteria rendered the highest specificity of 85%.

The highest positive LR among the single criteria, 3.92 (95% CI: 2.38–6.96), was seen for short axis size ≥ 5 mm, and among the combined criteria, the highest positive LR of 3.52 (95% CI: 2.18–5.85) was seen for the ESGAR consensus criteria.

The lowest negative LR among the single criteria, 0.32 (95% CI: 0.15–0.58), was seen for heterogeneous signal and among the combined criteria, the lowest negative LR of 0.24 (95% CI: 0.11–0.42) was seen for the combination of size ≥ 5 mm or size < 5 mm and either round shape or irregular margin.

The sensitivity, specificity, and positive and negative LR for the 2016 ESGAR consensus criteria when round shape was removed as a constituent morphological criterion were 81%, 51%, 1.66 (95% CI: 1.34–2.1), and 0.37 (95% CI: 0.19–0.65), respectively. The sensitivity, specificity, and LRs were similar when performing the same calculations for the 30 patients (340 mesorectal nodal structures) with early/intermediate disease, as demonstrated in Supplemental Tables 4 and 5.

## Discussion

In this anatomically matched lesion-by-lesion study of rectal cancer specimens, we could show that it was relatively common for nodal structures assessed as lymph nodes on MRI to prove to be TDs and, to a lesser extent, nodular EMVI histopathologically. We also could show a statistically significant univariable association between the 2016 ESGAR consensus criteria and a malignant nodal outcome, as well as for all the individual criteria that constitute the ESGAR consensus criteria, except for round shape. The round shape also had a positive LR of 1.49 and a high negative LR close to unity, which indicates that this criterion might be of low diagnostic value. Although round shape probably is not used clinically with as strict a definition as we used in this study, one might have to reconsider its usefulness. However, removing the round shape as a constituent morphological criterion from the 2016 ESGAR consensus criteria still rendered a low specificity of 51% and a low positive LR of 1.66, although the negative LR improved from 0.54 to 0.37 and the sensitivity from 54% to 81%. In the latest attempt to address the lack of consensus in N-staging in general, a scoring system called Node Reporting and Data System 1.0 (Node-RADS), a round shape is defined as a spherical form without fatty hilum, which could be interpreted as the presence of a round shape in two or more planes and thus might be a way forward in utilising this criterion [16]. In all other aspects, Node-RADS for mesorectal nodal structures are similar to the ESGAR consensus criteria; a nodal structure suspicious for malignancy according to the consensus criteria would render a Node-RADS score of high suspicion level for malignancy [16].

In our data, the 2016 ESGAR consensus criteria demonstrated a lower sensitivity whilst a higher specificity than the pooled sensitivity and specificity from the recent meta-analysis by Zhuang et al: 54% and 85%, respectively, compared to 73% and 74%, respectively [9]. However, only 5 of 36 of the included studies in the meta-analysis, all published before 2015 [6, 17–21], had an individual nodal anatomical matching between imaging and histopathology. When Zhuang et al performed a subgroup analysis on these five studies, the sensitivity and specificity were 55% and 89%, respectively, which is similar to our results.

Furthermore, a large Dutch study from 2018 that included 30 161 rectal cancer patients showed an even lower sensitivity of 38% than presented in our study but a higher specificity of 87% for N-stage assessment in rectal patients who did not receive neoadjuvant treatment and a sensitivity and specificity of 56% and 67% for patients who received such treatment [17]. In the above study, the criteria for malignancy changed several times during the inclusion period from 2003 to 2014, thus demonstrating within-study heterogeneity [17]. Thus, the available literature highlights the existing challenge with MRI for assessment of the N-stage in rectal cancer: the difference in the criteria used by radiologists to assess N-stage and the inability of existing criteria to appropriately evaluate N-stage with MRI [5, 17]. This also indicates a clinical challenge, as the contemporary N-staging assessment might not be accurate enough to properly inform recommendations for neoadjuvant treatment. In the 2017 European Society for Medical Oncology (ESMO) clinical practical guidelines for rectal cancer, mesorectal lymph node metastases are considered less critical mainly due to the challenges with N-stage assessment [4]. In our study, 44% of the matched nodal structures, initially assessed as mesorectal lymph nodes, were histopathologically proven to be TDs or nodular EMVI, regardless of early/intermediate disease (PS group) or more advanced disease (NT group). Since both TDs and EMVI are associated with a poorer prognosis than malignant lymph nodes alone [5–8], this might indicate that nodal staging in general, and differentiating between the nodal entities in particular, still is relevant. However, the question remains on how to handle the challenges with a less accurate N-staging clinically.

As mentioned earlier, a higher sensitivity than in the earlier meta-analyses could be seen when removing round shape from the 2016 ESGAR consensus criteria, but at the expense of specificity. The most important feature depends on the clinical setting, where a high sensitivity to correctly identifying a disease (e.g. nodal malignancy) is preferable for ruling out disease, for example, when deciding on neoadjuvant treatment to avoid potential overtreatment [18]. On the other hand, high specificity to correctly identify a lack of disease is preferable for ruling in disease, for example, before considering local excision approaches such as transanal endoscopic microsurgery or endoscopic submucosal/intramuscular dissection [4, 19]. Nevertheless, sensitivity and specificity alone may not always provide enough information to confirm (rule in) or exclude (rule out) disease. Instead, the more comprehensive information given by the LRs can be used in a similar manner, where a high positive LR can be used for ruling in diseases since it increases the probability of disease, and a low negative LR can be used for ruling out disease since it lowers the probability of disease [20]. However, strong evidence for ruling in and out disease is typically for LRs above 10 and below 0.1, respectively [20]. In our data, the highest sensitivity of 91% was seen for one of the criteria combinations (size ≥ 5 mm vs size < 5 mm with either round shape or irregular margin), with a corresponding lowest negative LR of 0.24, which might not be enough to rule out disease. Only size ≥ 5 mm for ruling in disease approached a positive LR of 4, still short of certainty using the abovementioned rule.

The present study has some limitations. The meticulous data collection, where nodal lesions were matched across multiple MRI examinations to histopathology, resulted in a small sample size when considering individual patients and tumours, whilst the number of mesorectal nodal structures was higher. There is still some risk of inadequate statistical power to detect differences, as evidenced by the generally wide CIs for individual characteristics. This data collection process depended on a close collaboration between the study radiologist and the study pathologist over a long period of time, and the logistics could not be continued indefinitely, making it challenging to include more patients than already included. Moreover, the radiological findings were evaluated exclusively by the study radiologist with six years of experience in pelvic MRI, potentially introducing misclassification. Nevertheless, the initial calibration with a radiologist (L.B.) who is highly experienced in pelvic MRI and a subsequent calibration meeting during the evaluation period with both L.B. and another highly experienced radiologist (K.R.) might have mitigated this potential weakness. Furthermore, this is one of few studies where precise anatomical matching has been done to identify individual lymph nodes and other nodal structures. This is a clear advantage when assessing the ESGAR criteria.

Another limitation was the exclusion of the histopathologically benign nodal structures in the group that received neoadjuvant treatment. Since malignant nodal structures were generally larger than benign, this selection of patients favoured the selection of larger malignant nodal structures in our cohort. Although the same statistically significant strong association between size and malignant outcome was seen when assessing the upfront surgery group only, the matched nodal structures were still significantly larger than the unmatched. Thus, these methodological considerations in our specific cohort might influence the observed strong association between short-axis size and malignant outcome. For this reason, strong conclusions regarding the importance of size for the prediction of malignancy cannot be drawn. However, as demonstrated in Fig. 2, it was clear that the proportion of malignant nodal structures increased with size, which also is consistent with previous studies [21, 22]. Furthermore, there might be biological differences between patients selected for neoadjuvant treatment and those receiving upfront surgery. Still, only the baseline MRI was used to assess malignant characteristics, and statistically, as mentioned earlier, the results were similar when evaluating only nodes in the primary surgery group. Lastly, the 2016 ESGAR consensus criteria are supposed only to be used to assess mesorectal lymph nodes. However, in our material, some nodal structures assessed as lymph nodes on baseline MRI proved to be TDs and even nodular EMVI. We chose not to exclude those nodal structures since this is the reality in which radiologists work today. There are currently no criteria to discriminate mesorectal lymph nodes from TDs or nodular/discontinuous presentation of EMVI. In addition, the definition of TDs has changed over time. When Brown et al introduced morphological criteria in 2003, all malignant nodules over the size of 3 mm were classified as metastatic lymph nodes regardless of whether remnant lymphoid tissue could be detected, as defined in TNM 5 [23]. In the current TNM classification (TNM 8), TDs are defined as discrete nodules of tumours in the perirectal fat without histological evidence of residual lymph nodes or identifiable vascular or neural structures [24]. In Brown et al’s article, 57% of the metastatic lymph nodes with uneven contour lacked remnant lymphoid tissue. Today, many of these lesions would probably have been classified as TDs, not lymph nodes.

**Fig. 3:**
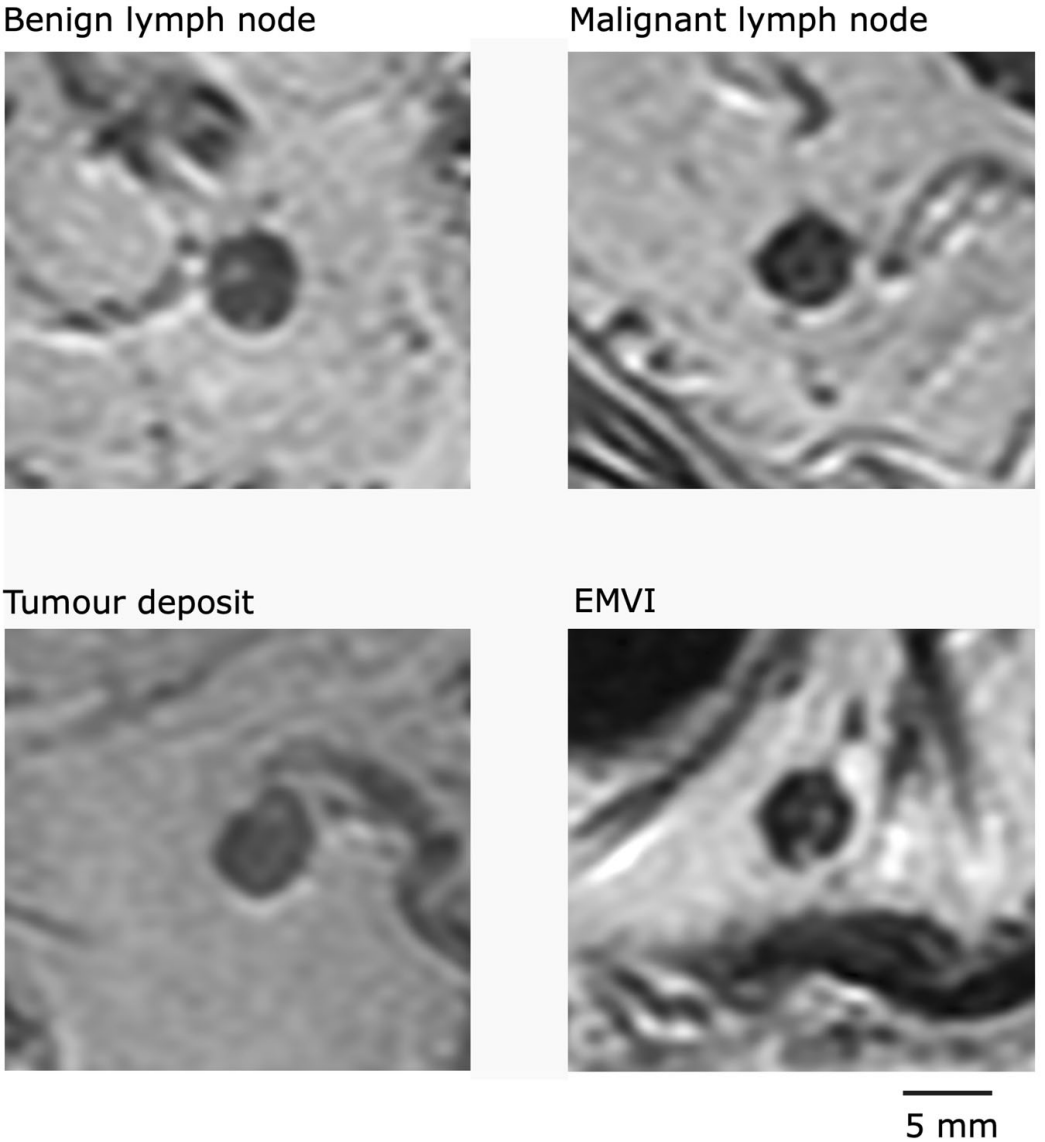
Examples of four anatomically matched nodal structures, all with a short axis size of 5.0 mm

As mentioned earlier, radiologists’ difficulties in visually separating lymph nodes from TDs and even nodular EMVI, as demonstrated in Fig. 3, pose an important challenge [8]. In this study, we investigated the association between the comet tail appearance and malignant outcome. Lord et al proposed this criterion as a sign of TDs in the ongoing COMET trial [14].

Due to the low number of nodal structures presenting with the criteria and only 36% of those nodal structures that proved to be TDs, this study can only suggest that further research is needed to investigate the correlation between the comet tail appearance and TDs. Unfortunately, there seems to be high interobserver variability among pathologists when attempting to separate lymph nodes from TDs, thus adding an additional level of difficulty in creating reliable radiological criteria [17, 25, 26].

## Conclusion

This prospective anatomically matched study demonstrates a positive association between the 2016 ESGAR consensus criteria and its constituent features with malignant nodal outcomes. However, the combination of morphological criteria and size, as used in the 2016 ESGAR consensus criteria, might not be accurate enough for predicting malignancy in lymph nodes in rectal cancer, although some criteria or combinations may be useful for indicating a very low risk of lymph node involvement. Nevertheless, the generally low diagnostic performance of both individual and combined criteria and the inability to discriminate lymph nodes from other malignant nodal structures continue to make contemporary nodal staging challenging in rectal cancer patients.

## Supplementary information

ELECTRONIC SUPPLEMENTARY MATERIAL

## Abbreviations

CI: Confidence interval
CT: Computerised tomography
EMVI: Extramural venous invasion
ESGAR: European Society of Gastrointestinal and Abdominal Radiology
FDG: ^18^F-fluoro-2-deoxy-d-glucose
LR: Likelihood ratio
MRI: Magnetic resonance imaging
PET: Positron-emission tomography
RECTOPET: Rectal cancer trial on PET/MRI/CT
TD: Tumour deposit
