# Supplementary material for: Evaluation of MRI characterisation of histopathologically matched lymph nodes and other mesorectal nodal structures in rectal cancer
Source: Eur Radiol. 2025 Jan 21;35(7):4080–90. doi: 10.1007/s00330-025-11361-2 (PMC12165870; doi:10.1007/s00330-025-11361-2)
Supplement: Supplementary file 1 — ELECTRONIC SUPPLEMENTARY MATERIAL [file 330_2025_11361_MOESM1_ESM.pdf]

Evaluation of MRI characterisation of histopathologically matched lymph nodes and other mesorectal nodal structures in rectal cancer.

ELECTRONIC SUPPLEMENTARY MATERIAL

Supp Table 1. MRI parameters used in the RECTOPET study.

Sequence	TE (ms)	TR (ms)	Field of view (mm)	Echo train length	Number of excitations	Slice thickness/gap (mm)	Matrix	Band-width (Hz/px)
**Sag T2 FRFSE**	102	3900	200 × 200	20	3	3.0/0.0	320x320	223
**Ax T2 FRFSE**	100	5719	270 × 270	16	1	4.0/0.4	384x256	325
**Cor T2 FRFSE**	102	4000	220 × 220	23	3	3.0/0.0	320x320	260
**T2 perpendicular (ax)**	100	4000	210 × 210	16	2	3.0/0.0	384x256	260
**Ax DWI Focus**	69.4	3500	240 × 120	–	1	4.0/0.0	160x180	3125
**Ax T1 FSPGR**	1.9	4.7	256 × 256	–	1	1	256x256	488

FRFSE: Fast relaxation fast spin echo,
FSPGR: Fast spoiled gradient echo,
TR: Repetition time,
TE: Echo time,
DWI: Diffusion-weighted magnetic resonance imaging

Supp Table 2. Description of all 609 matched and unmatched nodal structures found at baseline MRI and histopathological assessment according to short axis size and matching status for the 46 included patients – including the 151 excluded histopathological benign nodal structures in the neoadjuvant treatment group.

	Found at baseline MRI			Found at histopatology		
	Matched	Unmatched	p-value	Matched	Unmatched	p-value
Median short axis size in mm (IQR)	3.3 (2.4–4.7)	2.3 (1.7–3.2)	<0.001	2.7 (2.0–3.7)	1.7 (1.2–2.4)	<0.001
Nodal structures < 5 mm (%)	473 (78%)	531 (95%)		548 (90%)	1079 (99%)	
N-	502 (82%)			502 (82%)	998 (92%)	
N+	60 (9.9%)			60 (9.9%)	44 (4.0%)	
TD	39 (6.4%)			39 (6.4%)	34 (3.1%)	
EMVI	8 (1.3%)			8 (1.3%)	12 (1.1%)	
Total number	609	560		609	1088	

N-= benign lymph nodes, N+= malignant lymph nodes, TD= tumour deposits, EMVI= extramural venous invasion
IQR= Interquartile range

Supp Table 3. This includes the primary surgery group only, consisting of 27 patients and 369 node by node matched mesorectal lymph nodes. Univariable and multivariable odds ratios (ORs) with 95% confidence intervals (CIs) in predicting histopathological malignancy for different MR-morphological characteristics in mesorectal nodal structures. The individual variables used for the multivariable model are the constituent criteria in the ESGAR consensus criteria, i.e., irregular margin, round shape, heterogeneous signal and size.

Radiological characteristics	N-	N+	Univariable model			Multivariable model		
	n (%)	n (%)	OR	95% CI	p value	OR	95% CI	p value
**Irregular margin**								
No	150 (98.0%)	3 (2.0%)	1.00			1.00		
Yes	201 (93.0%)	15 (7.0%)	5.46	1.22–24.45	0.026	1.12	0.18–6.85	0.906
**Round shape**								
No	318 (95.8%)	14 (4.2%)	1.00			1.00		
Yes	33 (89.2%)	4 (10.8%)	3.60	0.74–17.37	0.111	2.01	0.32–12.63	0.458
**Comet tail appearance***								
No	341 (95.8%)	15 (4.2%)	1.00					
Yes	10 (76.9%)	3 (23.1%)	4.96	0.70–35.02	0.108			
**Heterogeneous signal**								
No	142 (99.3%)	1 (0.7%)	1.00			1.00		
Yes	209 (92.5%)	17 (7.5%)	10.9	1.24–95.85	0.031	5.13	0.46–57.78	0.186
**Short axis size**								
<5 mm	305 (97.1%)	9 (2.9%)	1.00			1.00		
≥5 mm	46 (83.6%)	9 (16.4%)	17.11	3.43–85.38	<0.0001	10.04	1.72–58.61	0.010

Supp Table 4. Radiological staging for determination of early/intermediate rectal cancer (defined as clinical stage T3b or less, no involvement of mesorectal fascia, and no presence of EMVI) for the included 46 patients.

Radiological staging	N (%)
**mrT**	
**2**	22 (47.8%)
**3a**	3 (6.5%)
**3b**	9 (19.6%)
**3c**	5 (10.9%)
**3d**	2 (4.3%)
**4a**	3 (6.5%)
**4b**	2 (4.3%)
**mrMRF+**	
**No**	37 (80.4%)
**Yes**	9 (19.6%)
**mrEMVI+**	
**No**	39 (84.8%)
**Yes**	7 (15.2%)
**Early/intermediate rectal cancer**	
**No**	16 (34.8%)
**Yes**	30 (65.2%)

MRF+: involvement of the mesorectal fascia
EMVI+: presence of extramural venous invasion

Supp Table 5. The sensitivity, specificity, positive and negative likelihood ratio with 95% confidence intervals for different criteria to predict malignancy in mesorectal nodal structures in early/intermediate rectal cancer (defined as clinical stage T3b or less, no involvement of mesorectal fascia, and no presence of EMVI).

	Sensitivity	Specificity	Positive likelihood ratio	Negative likelihood ratio
**Single criterion**				
Size ≥ 5mm	53%	87%	3.95 (1.33-8.35)	0.54 (0.22-0.92)
Irregular margin	85%	42%	1.48 (1.15–1.86)	0.35 (0.16–0.80)
Round shape	12%	92%	1.38 (0.00–2.94)	0.96 (0.84–1.09)
Heterogeneous signal	88%	41%	1.48 (1.21–1.77)	0.29 (0.08–0.68)
**Combined criteria**				
Size ≥ 5mm,<br>Size <5 mm with heterogeneous signal	88%	41%	1.48 (1.21–1.77)	0.29 (0.08-0.68)
Size ≥ 5mm,<br>Size <5 mm with irregular margin	85%	42%	1.47 (1.15–1.86)	0.35 (0.16–0.81)
Size ≥ 5mm,<br>Size <5 mm with round shape	59%	81%	3.05 (1.26–5.58)	0.51 (0.13–0.92)
Size ≥ 5mm,<br>Size <5 mm with round shape and irregular margin	53%	84%	3.24 (1.16–6.51)	0.56 (0.22–0.95)
Size ≥ 5mm,<br>Size <5 mm with either round shape or irregular margin	91%	39%	1.50 (1.22–1.81)	0.23 (0.00–0.63)
Size ≥ 5mm, Size <5 mm with round shape and heterogeneous signal	56%	84%	3.42 (1.33–6.25)	0.53 (0.20–0.90)
Size ≥ 5mm,<br>Size <5 mm with either round shape or heterogeneous signal	91%	38%	1.46 (1.18–1.71)	0.23 (0.00–0.71)
**ESGAR consensus criteria**	53%	85%	3.52 (1.28–7.22)	0.55 (0.22–0.94)
**ESGAR consensus criteria minus round shape**[a]	79%	50%	1.60 (1.18–2.12)	0.41 (0.15–0.75)

ESGAR: European Society of Gastrointestinal and Abdominal Radiology

[a] Defined as a short axis size of < 5 mm with heterogeneous signal and irregular margin, a short axis size of 5–8 mm with either heterogeneous signal or irregular margin, or a short axis size of > 9 mm.
